# Robust Self-Renewal of Rat Embryonic Stem Cells Requires Fine-Tuning of Glycogen Synthase Kinase-3 Inhibition

**DOI:** 10.1016/j.stemcr.2013.07.003

**Published:** 2013-08-22

**Authors:** Yaoyao Chen, Kathryn Blair, Austin Smith

**Affiliations:** 1 Wellcome Trust-Medical Research Council Stem Cell Institute, University of Cambridge, Tennis Court Road, Cambridge CB2 1QR, UK; 2 Department of Biochemistry, University of Cambridge, Tennis Court Road, Cambridge CB2 1QR, UK

## Abstract

Germline-competent embryonic stem cells (ESCs) have been derived from mice and rats using culture conditions that include an inhibitor of glycogen synthase kinase 3 (GSK3). However, rat ESCs remain susceptible to sporadic differentiation. Here, we show that unsolicited differentiation is attributable to overinhibition of GSK3. The self-renewal effect of inhibiting GSK3 is mediated via β-catenin, which abrogates the repressive action of TCF3 on core pluripotency genes. In rat ESCs, however, GSK3 inhibition also leads to activation of differentiation-associated genes, notably lineage specification factors *Cdx2* and *T*. Lowered GSK3 inhibition reduces differentiation and enhances clonogenicity and self-renewal. The differential sensitivity of rat ESCs to GSK3 inhibition is linked to elevated expression of the canonical Wnt pathway effector LEF1. These findings reveal that optimal GSK3 inhibition for ESC propagation is influenced by the balance of TCF/LEF factors and can vary between species.

## Highlights

• Rat ESCs are susceptible to sporadic differentiation
• High GSK3 inhibition induces lineage specification genes
• Reduced GSK3 inhibition derepresses pluripotency factors via TCF3
• Threshold sensitivity is due to high LEF1, which activates differentiation genes

## Graphical Abstract

**Figure:**
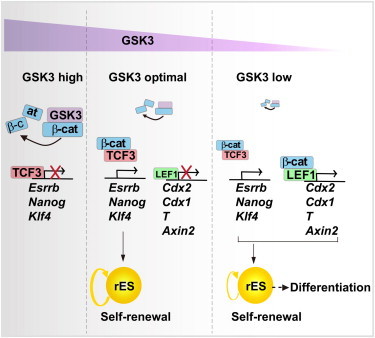

## Introduction

The degree to which embryonic stem cells (ESCs) represent generic properties of pluripotent founder cells in mammalian embryos is unresolved (Nichols and Smith, 2012; Smith, 2001). Mouse ESCs are the paradigmatic model. However, it is increasingly clear that there are differences in early embryos and derivative stem cells among mammals (Nichols and Smith, 2009; Roode et al., 2012; Rossant, 2008). In this context, rat ESCs provide a useful comparator for interrogating pluripotency in vitro and seeking to extract generic principles.

ESCs from mouse and rat can be derived and maintained using the cytokine leukemia inhibitory factor (LIF) in combination with two small molecule inhibitors (2i) that block the mitogen activated protein kinase (MAPK/ERK) pathway and reduce the activity of GSK3 (Buehr et al., 2008; Kawamata and Ochiya, 2010; Li et al., 2008; Nichols et al., 2009b; Ying et al., 2008). Rat ESCs can colonize chimeras and pass through the germline, thereby fulfilling the functional criteria for naive pluripotent stem cells. However, rat ESCs differ from mouse ESCs in a propensity to undergo unscheduled differentiation, which can lead to complete collapse of cultures (Blair et al., 2012).

The interplay between extrinsic regulators and the transcriptional circuitry that governs pluripotent stem cell self-renewal is incompletely understood (Chen et al., 2008; Niwa, 2007). Mouse and rat ESCs appear to express similar core pluripotency factors that are central to establishing and maintaining the naive pluripotent state (Blair et al., 2011). However, rat ESCs also express lineage determination factors (Hong et al., 2013) that are suppressed in mouse ESCs cultured in 2i (Marks et al., 2012). Here, we explore the inappropriate expression of lineage-specifying transcription factors in undifferentiated rat ESCs. We reveal an underlying mechanism that can be counteracted to stabilize self-renewal.

## Results

### Undifferentiated Rat ESCs Exhibit Ectopic Expression of *Cdx2*

**Figure 1:**
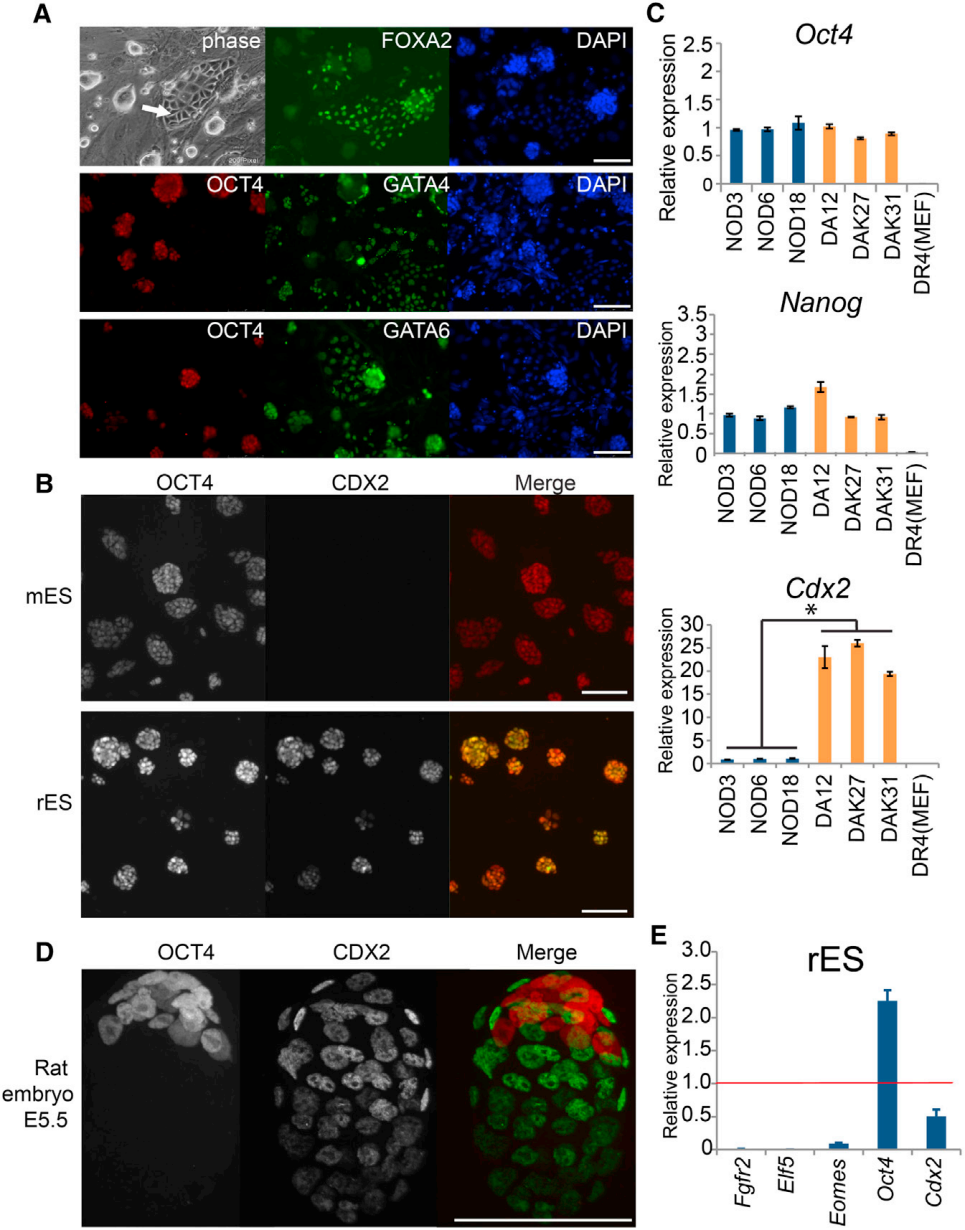
Differentiation and Ectopic Expression of CDX2 in Rat ESCs (A) Bright-field and immunofluorescence images of rat ESCs in 2iL on feeders. (B) Immunostaining of OCT4 and CDX2 in mouse (mES) and rat (rES) ESCs. (C) Comparative analysis by qRT-PCR of *Oct4*, *Nanog*, and *Cdx2* transcripts in mouse (blue) and rat (orange) ESCs using primers designed against conserved sequences. Expression values are normalized to *Gapdh* and relative to the average of mouse samples. Data were analyzed by unpaired t test. ^∗^p < 0.01. (D) Immunostained rat E5.5 blastocyst. (E) qRT-PCR analysis of *Fgfr2*, *Elf4*, *Eomes*, *Oct4*, and *Cdx2* in rESCs in 2iL (blue) and rat embryonic day 5.5 (E5.5) whole blastocysts (red line). Values are normalized to *Gapdh* and relative to the average in rat blastocysts. Error bars are SD of technical triplicates. Scale bar, 100 μM.

Mouse ESCs cultured in 2i with LIF (2iL) are relatively homogeneous with negligible transcription of most differentiation-affiliated genes and no overt differentiation (Marks et al., 2012; Wray et al., 2010). In contrast, overtly differentiated cells are frequently observed in rat ESC cultures. These flattened cells generally emerge sporadically around the perimeter of undifferentiated colonies but can expand independently (Figure 1A, arrow) such that they tend progressively to dominate cultures. These differentiated cells are immunopositive for GATA4, GATA6, and FOXA2 (Figure 1A), suggesting a primitive or definitive endoderm identity. Rat extraembryonic stem cells have previously been reported to express CDX2 (Buehr et al., 2003; Chuykin et al., 2010; Galat et al., 2009). We found that CDX2 protein was not detected in overtly differentiated cells. Unexpectedly, however, CDX2 was coexpressed with OCT4 in the majority of undifferentiated cells (Figure 1B). *Cdx2* expression was confirmed by quantitative RT-PCR (qRT-PCR) (Figure 1C). We then compared three mouse ESC lines (NOD3, NOD6, and NOD18) and three rat ESC lines (DA12, DAK27, DAK31) and found that while the transcript levels of pluripotency factors such as *Oct4* and *Nanog* are comparable, the expression of *Cdx2* is 20-fold higher in the rat lines (p < 0.01).

In the mouse embryo, CDX2 becomes confined to trophectoderm during blastocyst formation (Beck et al., 1995). OCT4 and CDX2 are thought to become mutually exclusive via reciprocal repression (Niwa et al., 2005). We used immunostaining to examine whether this relationship is conserved in rat blastocysts and indeed found that CDX2 is restricted to the trophoblast while OCT4 marks the inner cell mass (Figure 1D). The contrast between mutually exclusive expression of CDX2 and OCT4 in the blastocyst and coexpression in rat ESCs indicates that the presence of CDX2 is an aberrant property acquired in vitro. The abundance of CDX2 transcript and protein in rat ESCs could potentially trigger trophoblast differentiation (Niwa et al., 2005). However, key trophectodermal lineage markers *Fgfr2*, *Elf5*, and *Eomes* were barely detectable by qRT-PCR in rat ESCs (Figure 1E). Thus, expression of CDX2 in rat ESCs does not reflect trophoblast priming. However, CDX2 is expressed in other lineages and might destabilize the pluripotency network.

### *Cdx2* Expression in Rat ESCs Is Induced by GSK3 Inhibition

The zebrafish homolog of mammalian *Cdx2*, *cdx1a*, has been shown to respond to Wnts (Shimizu et al., 2005). GSK3 inhibition has pleiotropic effects that include simulation of canonical Wnt signaling through stabilizing β-catenin (Doble and Woodgett, 2003). Notably, inhibition of GSK3 promotes endodermal differentiation in human ESCs (Bone et al., 2011). We therefore investigated whether GSK3 inhibition may contribute to aberrant gene expression and differentiation in rat ESC cultures.

**Figure 2:**
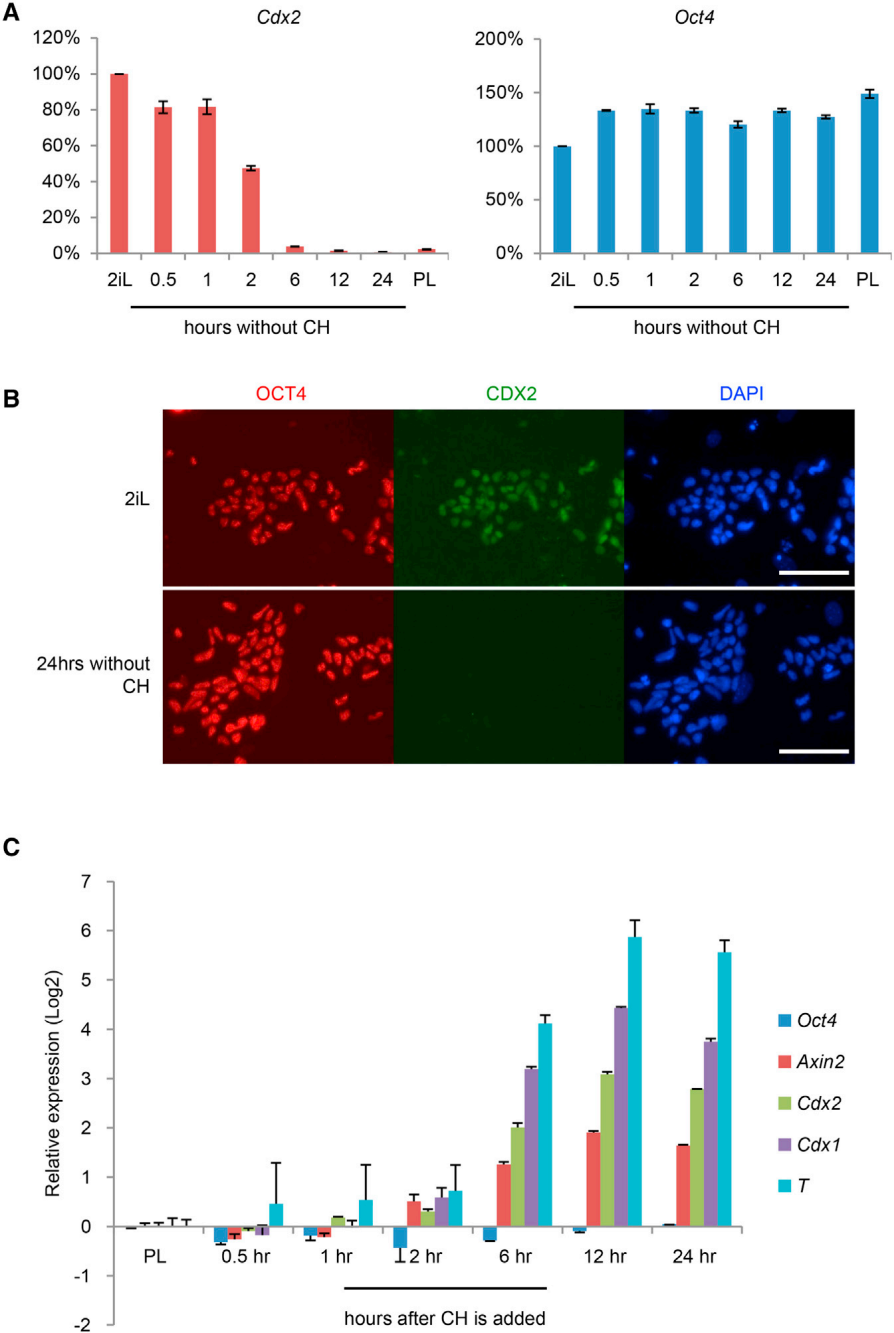
Effect of GSK3 Inhibition on *Cdx2* Expression (A) Expression of *Cdx2* and *Oct4* upon CH removal. Values are normalized to *Gapdh* and relative to 2iL. (B) Immunofluorescence for CDX2 and OCT4 in rat ESCs cultured in 2iL and 24 hr after CH removal. (C) Transcriptional response of rat ESCs to CH. Expression is normalized to *Gapdh* and relative to values in PL. Error bars are SD of technical triplicates. Scale bar, 100 μM.

We first monitored the response of *Cdx2* to withdrawal of the GSK3 inhibitor CHIR99021 (CH). As shown in Figure 2A, *Cdx2* messenger RNA (mRNA) fell within 30 min and by 24 hr had decreased to 1% of the level in 2iL. CDX2 protein was no longer detectable after 24 hr without CH (Figure 2B). In contrast, *Oct4* expression was fully maintained. We then examined the effect of reintroducing CH to rat ESCs. As shown in Figure 2C, while *Oct4* mRNA remained constant, the expression of *Cdx2* increased robustly over the 24 hr period following readdition of CH. Significantly, three canonical Wnt/β-catenin target genes, *Cdx1*, *Axin2*, and *T* (*brachyury*), followed a similar pattern. These data suggest GSK3 inhibition may be responsible for aberrant expression of differentiation genes in rat ESC cultures.

### Titration of GSK3 Inhibition Enhances Rat ESC Self-Renewal

CH promotes self-renewal of mouse ESCs primarily via the derepression of pluripotency genes that are bound by TCF3, notably *Nanog*, *Klf2*, and in particular *Esrrb* (Martello et al., 2012; Wray et al., 2011; Yi et al., 2011). Importantly, the effective concentration of CH has been empirically determined as 3 μM, which causes only partial inhibition of GSK3 (Ying et al., 2008). This concentration appears optimal for all mouse ESC lines cultured in 2i, with or without LIF and feeders (Nichols et al., 2009a). Under these conditions, *Esrrb* is fully induced but only modest activation of canonical Wnt target genes is evident (Martello et al., 2012; Wray et al., 2011). We investigated the possibility that the level of GSK3 inhibition might differentially affect the derepression of pluripotency factors versus the induction of canonical Wnt target genes in rat ESCs.

**Figure 3:**
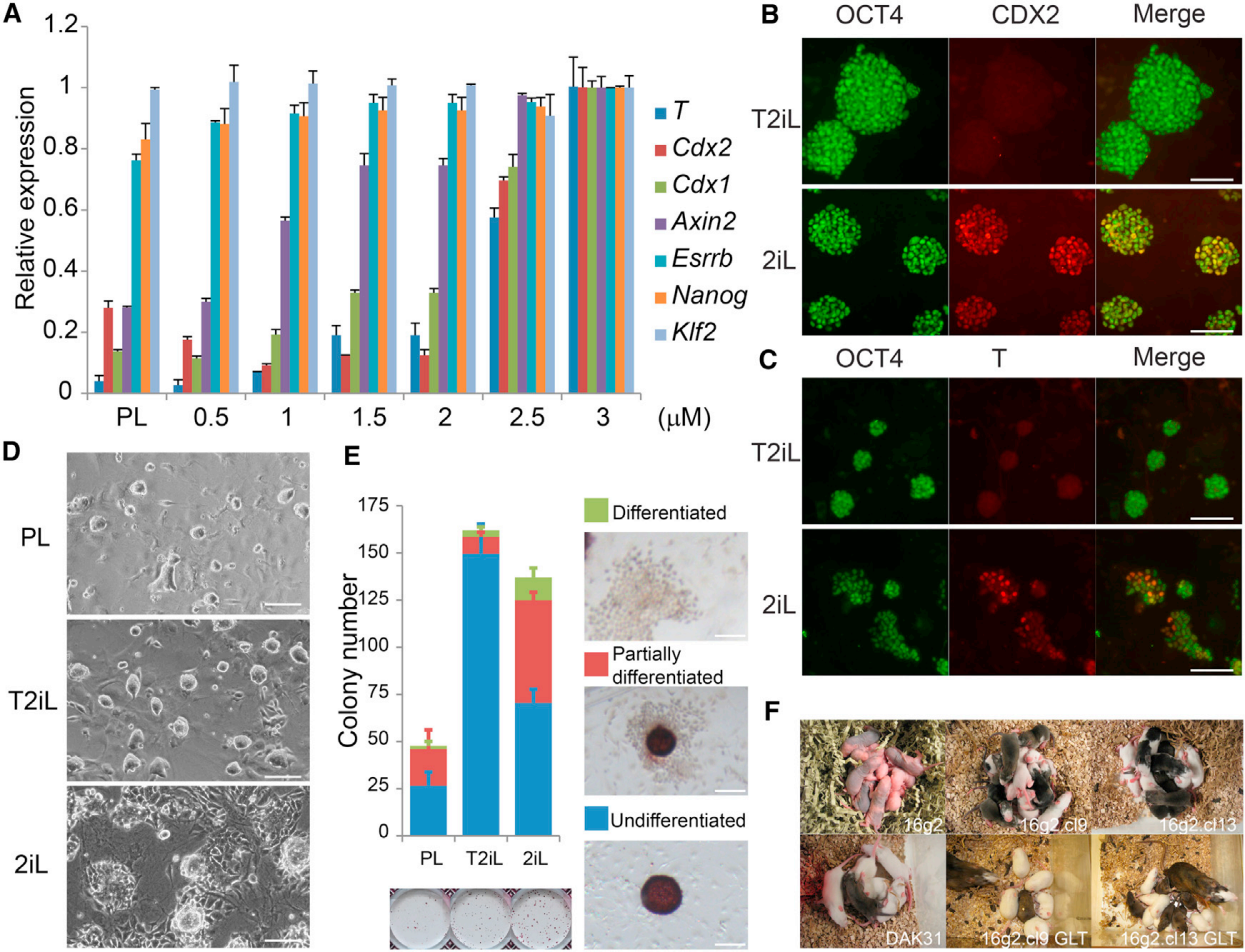
Titration of GSK3 Inhibition (A) qRT-PCR analysis of gene expression in rat ESCs cultured with different concentrations of CH. Values are normalized to *Gapdh* and relative to 2iL. Error bars represent SD of three technical replicates. (B and C) Immunofluorescence staining of rat ESCs cultured in T2iL or 2iL for CDX2 and T, respectively. (D) Morphology of rat ESC bulk cultures. (E) Colony formation from 250 single cells analyzed by AP staining. Error bars represent SD of four technical replicates. (F) Chimeras and germline F1 pups from injection of DA (Agouti) rat ESCs into SD (albino) blastocysts. Scale bar, 100 μM.

We propagated rat ESCs in PD03 and LIF (PL) for 8 days. CH was then added over a range from 0.5 to 3.0 μM. After 48 hr, cultures were harvested and analyzed for expression of *Cdx2*; canonical Wnt targets *Axin2*, *Cdx1*, and *T*; and TCF3-repressed pluripotency genes *Esrrb*, *Nanog*, and *Klf2* (Figure 3A). While the relative expression of *Axin2*, *Cdx1*, *T*, and *Cdx2* increased dramatically at higher levels of CH (Figure 3A), the expression of *Nanog*, *Klf2*, and *Esrrb* was less affected. Indeed, the pluripotency factors were all appreciably expressed in the absence of CH, possibly due to the influence of feeder cells. *Esrrb* and *Nanog* levels did increase in CH, but reached peak levels at only 1–1.5 μM. At 1 μM CH, differentiation genes are barely induced. Absence of both CDX2 and T proteins in this condition was confirmed by immunostaining (Figure 3B and 3C). We therefore selected 1 μM CH for further evaluation as a titrated 2iL (T2iL) condition.

We compared rat ESCs cultured in PL, T2iL, or 2iL for over four passages. Differentiated cells apparent in 2iL were not observed in T2iL or PL (Figure 3D). As a more rigorous examination of ability to support rat ESC propagation, we assayed colony formation from dissociated cells plated at low density. Colony forming efficiency was less than 20% in PL but greater than 50% in T2iL and 2iL (Figure 3E). Colonies appeared on average slightly larger in 2iL. However, alkaline phosphatase (AP) staining revealed that this was largely attributable to differentiated cells around the periphery of 2iL colonies (Figure 3E). In contrast there was little differentiation evident in T2iL colonies, consistent with observations on bulk culture. These results suggest that T2iL supports an increased frequency of self-renewal with reduced differentiation in rat ESC cultures.

To test whether T2iL maintains developmental identity and pluripotency, we assessed the ability of rat ESCs cultured in T2iL to colonize the developing embryo. A Dark Agouti (DA) cell line, DAK31, and a GFP transgenic DA cell line, 16g2 (Blair et al., 2012) were expanded in T2iL for 13 passages before microinjection into Sprague-Dawley blastocysts. In addition, 16g2 cells were expanded from single cells in T2iL. Two out of seven clones retained a euploid chromosome count, similar to recovery of normal karyotypes in previous clonal studies in 2iL (Blair et al., 2012; Tong et al., 2010). These two clones were injected and yielded coat-color chimeras as did the parental bulk culture. Both of the clones proved to be germline competent upon mating (Figure 3F; Table S1 available online). These data demonstrate that T2iL maintains full developmental competence.

### Rat ESCs Express LEF1 at Similar Levels to TCF3

CH is predicted to inhibit mouse and rat GSK3 with comparable efficiency because the proteins are near identical. Therefore, we explored potential differences downstream of GSK3. In mouse ESCs, β-catenin directly abrogates the function of TCF3, which acts on pluripotency factor genes as a transcriptional repressor (Cole et al., 2008; Pereira et al., 2006; Wray et al., 2011; Yi et al., 2011). However, β-catenin can potentially upregulate canonical Wnt pathway targets such as *Cdx1*, *Axin2*, *T* and, in rat, *Cdx2*. This could also involve removal of *Tcf3* repression and/or direct transcriptional activation involving other TCF/LEF family members (Yi et al., 2011).

**Figure 4:**
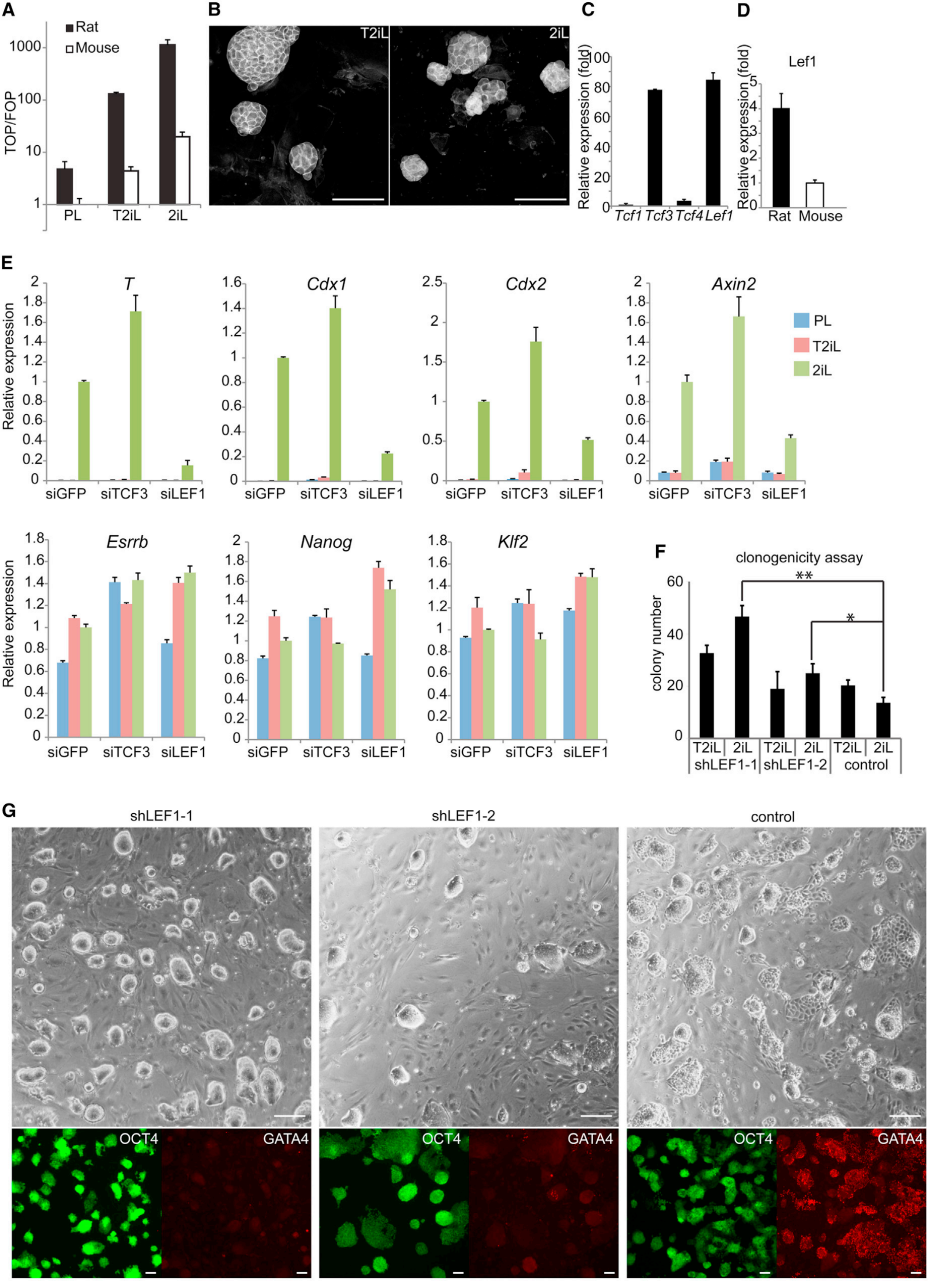
Interrogating Downstream Effectors of GSK3 Inhibition in Rat ESCs (A) TopFlash assay of β-catenin transcriptional activity in rat and mouse ESCs in PL, T2iL, and 2iL. Values are normalized to mouse ESCs in PL. Error bars represent SD of technical triplicates. (B) Immunostaining of β-catenin in rat ESCs cultured in T2iL and 2iL. (C) qRT-PCR analysis of *Tcf1*, *Tcf3*, *Tcf4*, and *Lef1* in rat ESCs maintained in T2iL, using TaqMan probes. Values are normalized to *Gapdh* and relative to *Tcf1*. (D) qRT-PCR analysis using conserved primers of *Lef1* expression in rat and mouse ESCs maintained in 2iL. (E) qRT-PCR analysis of a panel of gene expression after *Tcf3* and *Lef1* knockdown. Gene expression was normalized to *Gapdh* and relative to values in siGFP transfected cells cultured in 2iL. Error bars represent SD of technical triplicates. (F) Effect of stable knockdown of LEF1 on colony formation in 2iL or T2iL. A total of 80 cells were plated per well and analyzed by AP staining after 5 days. Error bars are SDs of four technical replicates. Data were analyzed by unpaired t test. ^∗^p < 0.01; ^∗∗^p < 0.001. (G) Bright-field and immunofluorescence images of rat ESCs in 2iL with or without *Lef1* knockdown. Scale bar, 100 μM.

We investigated β-catenin-mediated transcriptional activity using the TOPflash reporter assay (Molenaar et al., 1996). In the absence of CH, mouse ESCs on feeders show low activation of TOPFlash (Pereira et al., 2006; Wray et al., 2011). In contrast, rat ESCs showed appreciable TOPFlash activation in identical conditions (Figure 4A). In CH, both mouse and rat ESCs showed increased reporter activity, but rat cells showed a steeper dose-response curve reaching 60-fold greater TOPFlash activation in 2iL. Notably, however, the TOPFlash signal was 9-fold lower in T2iL than in 2iL. Consistent with this, intracellular β-catenin was readily apparent in rat ESCs in 2iL, but was faint in T2iL (Figure 4B). These data indicate that rat ESCs have a heightened canonical β-catenin-mediated transcription response to GSK3 inhibition that can be tempered by reducing the concentration of CH.

In mouse ESCs, TCF3 dominates over other TCF/LEF factors and constrains TOPFlash activity (Pereira et al., 2006; Wray et al., 2011; Yi et al., 2011). We measured mRNA levels of *Tcf/Lef* family members in rat ESCs. *Lef1* mRNA is expressed at a high level similar to *Tcf3* (Figure 4C). Using primers against conserved sequences, we confirmed that *Lef1* transcript level is more abundant in rat than mouse ESCs (Figure 4D). We also observed that *Tcf3* expression is downregulated by CH while *Lef1* shows the reverse relationship (Figure S1A).

We used small interfering RNA (siRNA) to knock down *Tcf3* or *Lef1* in rat ESCs. Rat ESCs transfected with siRNAs were cultured in PL, T2iL, or 2iL for 48 hr, followed by qRT-PCR analysis. *Tcf3* knockdown resulted in upregulation of *Esrrb* and to a lesser extent of *Nanog* and *Klf2*, which was not augmented by addition of CH (Figure 4E). In contrast, these genes were not affected by *Lef1* siRNA. Therefore, the effect of GSK3 inhibition on pluripotency genes is mediated principally through elimination of TCF3 repression, as in mouse ESCs (Martello et al., 2012). Notably, depletion of *Tcf3* transcript did not impair the induction by CH of *Cdx2* and established Wnt targets. However, these genes showed a significantly reduced response after siLEF1 transfection. Expression of *T* in 2iL fell by more than 80% in siLEF1-treated cells and the other markers were reduced by 70%–50% (Figure 4E).

Finally, we investigated whether stable *Lef1* knockdown may reduce the susceptibility of rat ESCs to differentiation. We introduced two small hairpin RNAs (shRNAs; shLEF1-1 and shLEF1-2) into rat ESCs using piggyBac transposon vectors (Figure S1B). The vectors also contain a GFP cassette, allowing enrichment for expression by flow cytometry. Knockdown efficiency was higher for the shLEF1.1 construct (Figure S1C), and this yielded substantially increased numbers of undifferentiated colonies in both T2iL and 2iL, with more colonies in the latter condition (Figure 4F). shLEF1.2 transfectants showed the same trend but to a lesser extent. Furthermore, bulk-cultured shLEF1-1 and shLEF1-2 cells remained morphologically undifferentiated and GATA4 negative after four continuous passages in 2iL, while differentiation was evident in the control (Figure 4G). These findings support the hypothesis that sensitivity of rat ESCs to CH-induced expression of differentiation genes is largely attributable to the abundance of LEF1.

## Discussion

Rat ESCs can be derived with similar high efficiency to mouse ESCs using 2i with LIF, yet they are more prone to differentiation during expansion (Blair et al., 2011). The present findings indicate that differentiation is triggered by overinhibition of GSK3 and can be suppressed by fine-tuning the inhibitor concentration. The altered sensitivity of rat ESCs appears to arise from higher expression of *Lef1*. However, different thresholds for β-catenin/TCF3-mediated derepression of pluripotency factors versus β-catenin/LEF1-mediated upregulation of differentiation factors enable precise titration of GSK3 inhibition to favor self-renewal.

Coexpression of CDX2 and OCT4 in rat ESCs is surprising. However, other trophectoderm lineage markers are not appreciably expressed and trophoblast-like cells are not seen. Therefore, CDX2 is not sufficient to activate a trophoblast differentiation program in rat ESCs, in contrast to findings from mouse ESC overexpression (Niwa et al., 2005). Furthermore, OCT4 levels are not significantly reduced by the presence of CDX2. This may be attributable to the presence of 2i. Alternatively, the reciprocal inhibition circuit between *Oct4* and *Cdx2* may be specific to mouse (Berg et al., 2011).

It should also be noted that CDX2 is not exclusively a trophoblast lineage marker, but is also expressed in endoderm and cardiac progenitors. Presence of CDX2 in rat ESCs may therefore reflect a more general activation of lineage-specifying factors, including the mesendoderm factor T. Interestingly, whereas CDX2 is present in the majority of OCT4-positive rat ESCs in 2i, albeit with some variation in levels, T protein is detected only in a minority of cells.

We surmise that the inhibition of GSK3 should be precisely tuned such that intracellular β-catenin levels are sufficient to remove TCF3 from chromatin (Shy et al., 2013) but not to engage appreciably with other TCF/LEF factors. With optimal inhibition, TCF3 targets that contribute to ESC self-renewal, such as *Esrrb*, are fully derepressed but activation of lineage specification genes is minimal. This model is consistent with findings of differential effects of *Tcf3* and *Tcf1* in mouse ESCs (Yi et al., 2011). Indeed, mouse ESC self-renewal efficiency declines at CH concentrations higher than 3 μM (Ying et al., 2008), and in elevated concentrations of CH, mouse ESCs show upregulation of *T*, *Cdx1*, and *Cdx2* (Figure S2). Notably, mouse ESCs totally deficient in GSK3 (Doble et al., 2007) can self-renew without CH but accompanied by continuous differentiation (Ying et al., 2008).

Our findings indicate that rat ESCs are more sensitive to GSK inhibition primarily because of the relative abundance of LEF1, which favors the activation of differentiation genes. Their collective expression may challenge and destabilize the self-renewal circuitry. The TOPFlash assays also suggest that there may be higher basal levels of intracellular β-catenin in rat ESCs. This may contribute to the reduced threshold of GSK3 inhibition for full derepression of *Esrrb*. Rat ESCs cultured in T2iL retain competence to form chimeras and give germline transmission, even after clonal expansion, with at least comparable efficiency to previous reports from our group and others using 2iL (Blair et al., 2012; Tong et al., 2010). By reducing differentiation, culture in T2iL may offer a more robust platform for expansion and genetic manipulation. However, T2iL does not improve karyotype stability, implying that there is additional selection pressure in the present culture milieu.

In conclusion, these findings demonstrate that the requirements for optimal self-renewal of naive pluripotent stem cells are subtly different between rodent species, although following common underlying principles. We speculate that such divergence constitutes the challenge facing efforts to derive true ESCs from livestock species and primates.

## Experimental Procedures

### Chimera Production

Blastocyst microinjection was carried out as described (Blair et al., 2012) using host blastocysts from the albino Sprague-Dawley strain and Dark Agouti strain ESCs. Chimeras were identified by mixed coat colour. Animal studies were approved by the UK Home Office and carried out in a designated facility.

### Cell Culture

Rat ESCs were maintained as described previously (Blair et al., 2012) on mitotically inactivated mouse embryo fibroblasts in N2B27 basal medium with MEK inhibitor PD0325901 (1 μM), GSK3 inhibitor CHIR99021 (3 μM or as specified), and human recombinant LIF (10 ng/ml, prepared in-house).

### Gene Expression Analysis by Quantitative Real-Time PCR

Total RNA was isolated using the RNeasy Kit (QIAGEN) and complementary DNA prepared using SuperScriptIII (Invitrogen) and 3′RACE adaptor primers. For rat embryos, a pool of 14 embryonic day 5.5 rat blastocysts was harvested and homogenized using QIAshredder (QIAGEN) prior to total RNA extraction. For real-time PCR, we used TaqMan Fast Universal Master Mix and TaqMan probes (Applied Biosystems) or Fast SYBR Green Master Mix and primers (Table S2).

### RNA Interference

Rat ESCs were transfected with siRNA at a final concentration of 40 nM using Dharmafect 1 (Dharmacon, cat. T-2001-01) and then replated at a concentration of 30,000 cells per well in 12-well plates. Cells were harvested 48 hr later for gene expression analysis. *Lef1* siRNA was obtained from QIAGEN (SI00280658). Tcf3 siRNAs were used as a mixture of two siRNAs obtained from QIAGEN (SI01444149) and Dharmacon (J-048614-10). siGFP was custom designed and obtained from Invitrogen (target sequence: 5′-TGAACTTCAGGGTCAGCTTGC-3′).

### Stable Knockdown of *Lef1* Using Short Hairpin RNA

Rat ESCs were transfected with PiggyBac-shLEF1 constructs (Figure S1B; Table S4) and transposase vector using lipofectamine 2000 (Invitrogen). Cells were plated at a concentration of 5 × 10^5^ cells per well in 12-well plates. Medium was changed after 8 hr of incubation. Cells were expanded for four passages in T2iL before flow sorting for the high GFP expression (top 2%). The sorted populations were briefly expanded and used for analysis.

## Supplemental Information

Document S1. Supplemental Experimental Procedures, Figures S1 and S2, Tables S1–S4
