# Supplementary material for: Robust Self-Renewal of Rat Embryonic Stem Cells Requires Fine-Tuning of Glycogen Synthase Kinase-3 Inhibition
Source: Stem Cell Reports. 2013 Aug 22;1(3):209–17. doi: 10.1016/j.stemcr.2013.07.003 (PMC3849254; doi:10.1016/j.stemcr.2013.07.003)
Supplement: Document S1. Supplemental Experimental Procedures, Figures S1 and S2, Tables S1–S4 [file mmc1.pdf]

Stem Cell Reports, Volume *1*

Supplemental Information

Robust Self-Renewal of Rat Embryonic Stem Cells Requires Fine-Tuning of Glycogen Synthase Kinase-3 Inhibition

Yaoyao Chen, Kathryn Blair, and Austin Smith

Inventory of supplementary information

Supplementary figures: 2

Figure S1. Transient and stable knockdown of LEF1. Related to Figure 4

Figure S2. Response of mouse ES cells to GSK3 inhibition. Mentioned in discussion

Supplementary Tables: 4

Table S1. Summary of injections of DA rat ES cells into SD host blastocysts. Related to Figure 3

Table S2. Primers and probes for real-time PCR. Related to Figure 1-4

Table S3. Primary antibodies for immunofluorescence staining. Related to Figure 1-4

Table S4. Short hairpin RNA sequence of shLEF1-1 and shLEF1-2. Related to Figure 4

Supplementary experimental procedures

Supplementary Figure 1

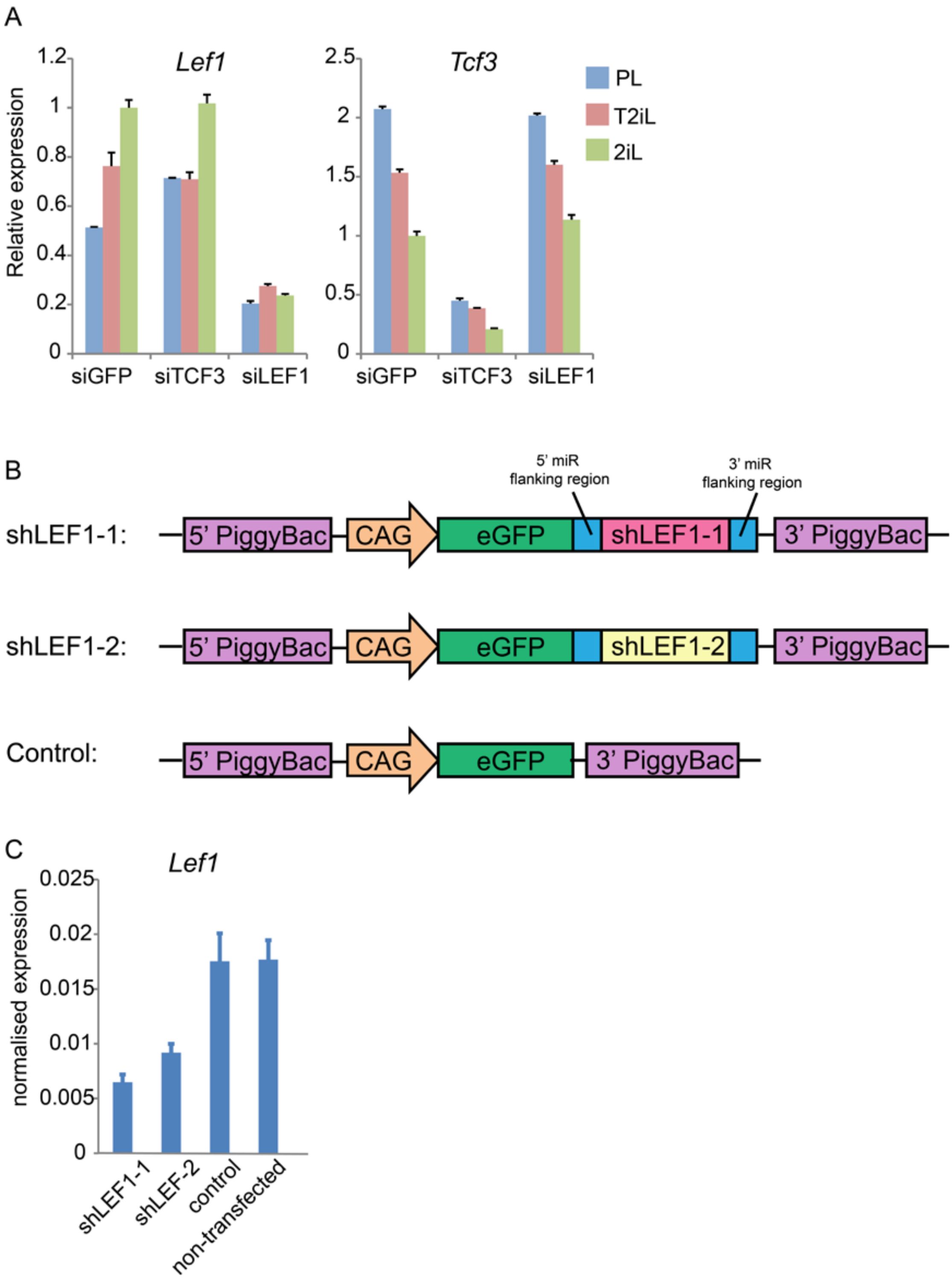

Figure S1. Transient and stable knockdown of LEF1. (A) qRT-PCR analysis of *Tcf3* and *Lef1* expression after *Tcf3* and *Lef1* knock down. Gene expression was first normalized to *Gapdh*, and then relative to values in siGFP transfected cells cultured in 2iL. (B) The design of PiggyBac vector for expression of *Lef1* shRNA. (C) qRT-PCR analysis of *Lef1* expression in

shLEF1 transfected cells versus control. Error bars represent standard deviation of three technical replicates.

Supplementary Figure 2

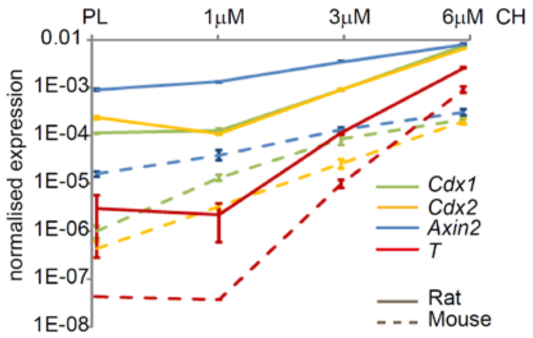

Figure S2. Response of mouse ES cells to GSK3 inhibition. qRT-PCR analysis of gene expression of *Cdx1*, *Cdx2*, *T*, and *Axin2* in mouse (dashed line) and rat (solid line) ES cells cultured on feeders with different concentrations of CH. Values are normalised to *Gapdh*. Error bars represent standard deviation of three technical replicates.

Table S1. Summary of injections of DA rat ES cells into Sprague-Dawley (SD) host blastocysts. Related to Figure 3

Cell line	Sex	Genetic modification	Passage number	Blastocysts transferred	Pups born	Chimaeras	Germline Transmission
16g2	F	GFP	35+**13**	25	16	10	0/4
DAK31	M	None	10+**13**	12	6	1	0/1
16g2.cl9	F	GFP	35+**12**	30	14	9	1/2
16g2.cl13	F	GFP	35+**12**	30	12	7	1/1

Passages in titrated 2i are labelled in bold.

Table S2. Primers and probes for real-time PCR.

Gene	Forward primer sequence	Reverse primer sequence
*Gapdh**	CAGTGATGGCATGGACTGTG	CAATGCATCCTGCACCAC
Esrrb	GGCGTTCTTCAAGAGAACCA	CCCACTTTGAGGCATTTCAT
*Nanog**	TACCTCAGCCTCCAGCAGAT	GCAATGGATGCTGGGATACT
Klf2	GGTAGTGGCGGGTAAGCTC	AACTGCGGCAAGACCTACAC
*Oct4**	CAGGGTCTCCGATTTGCAT	GCAGCTCAGCCTTAAGAACA
*Lef1**	CTGCTGTACATGTCACTGAAC	GATGGTGGCCTCTGTGTATG
*Cdx2**	AAGACAAATACCGGGTGGTG	CTGCGGTTCTGAAACCAAAT
Axin2(mouse)	GCAGGAGCCTCACCCTTC	TGCCAGTTTCTTTGGCTCTT
Cdx1(mouse)	ACGCCCTACGAATGGATG	CTTGGTTCGGGTCTTACCG
T(mouse)	CAGCCCACCTACTGGCTCTA	GAGCCTGGGGTGATGGTA

Gene	Company	Cat. No.
T(Rat)	Appliedbiosystem	Rn01527349_m1 (Cat#:4331182)
Axin2(Rat)	Appliedbiosystem	Rn00577441_m1 (Cat#:4331182)
Cdx1(Rat)	Appliedbiosystem	Rn01759334_m1 (Cat#:4331182)
Cdx2(Rat)	Appliedbiosystem	Rn00576694_m1 (Cat#:4331182)
Tcf1(Hnf1a) (Rat)	Appliedbiosystem	Rn00562020_m1 (Cat#:4331182)
Tcf3(Tcf7l1) (Rat)	Appliedbiosystem	Rn00483453_g1 (Cat#:4331182)
Tcf4(Rat)	Appliedbiosystem	Rn01411019_m1 (Cat#:4331182)
Lef1(Rat)	Appliedbiosystem	Rn01522501_m1 (Cat#:4331182)
Eomes(Rat)	Appliedbiosystem	Rn01746545_m1 (Cat. 4448892)
Elf5(Rat)	Appliedbiosystem	Rn01514160_m1(Cat. 4448892)
Fgfr2(Rat)	Appliedbiosystem	Rn01269940_m1(Cat. 4448892)

* Sequence is conserved between mouse and rat.

Table S3. Primary antibodies for immunofluorescence staining.

**Antigen**	**Species**	**Dilution**	**Company**	**Cat.No.**
CDX2	Rabbit	1:200	Cell signaling	3977S
OCT4(C-10)	Mouse	1:200	Santa Cruz	Sc-5279
T	Goat	1:200	R&D Systems	AF2085
CTNNB1 (β-catenin)	Mouse	1:400	BD Transduction Laboratories	610154
GATA4	Goat	1:100	Santa Cruz	sc-1237

Table S4. Short hairpin RNA sequence of shLEF1-1 and shLEF1-2. Related to Figure 4

shLEF1-1	GACTTGATGTCTGCTAAGTCGCGTTTTGGCCACTGACTGACGCGACTTAAGACATCAAGT
shLEF1-2	GTAATTGTCTCTCGCTGACCAGGTTTTGGCCACTGACTGACCTGGTCAGAGAGACAATTA

Supplementary experimental procedures

Immunofluorescence

Cells were fixed with 4% paraformaldehyde in PBS (pH 7.0) for 30 minutes at room temperature. Subsequently, cells were washed twice with PBST (0.1% Triton X-100 (Sigma) in 1XPBS) and then with blocking solution (4% donkey serum in PBST). Primary antibody solution was prepared by diluting antibody in blocking solution at the concentration listed in Table S3. Cells were incubated with the primary antibody at room temperature for 2 hours or at 4°C overnight, followed by three washes with Tris-buffered saline (TBS) containing 0.1% Tween 20 prior to incubation with the secondary antibodies at room temperature for 1 hour.

After nuclear staining with DAPI (Invitrogen), stained cells were detected by fluorescence microscopy or confocal-laser microscopy.

TOPFlash assay

10^6 cells per well were transfected with 4µg TOPFlash or FOPFlash (Upstate) and 0.2µg Renilla luciferase plasmids using lipofectamine 2000 (Invitrogen) in 6-well plates. Cells were passaged 24hrs later and replated on feeders in a 24 well plate in triplicates in PL, T2iL and 2iL respectively. 48hrs after transfection, cells were lysed and analysed using the dual luciferase kit (Promega) according to the manufacturer’s protocol.
